# Applicability of Yielding–Resisting Sand Column and Three-Dimensional Coordination Support in Stopes

**DOI:** 10.3390/ma12162635

**Published:** 2019-08-19

**Authors:** Fanbao Meng, Zhijie Wen, Baotang Shen, Yujing Jiang, Shaoshuai Shi, Renle Zhao

**Affiliations:** 1 State Key Laboratory of Mining Disaster Prevention and Control Co-founded by Shandong Province and the Ministry of Science and Technology, Shandong University of Science and Technology, Qingdao 266590, China; 2 Research Center of Geotechnical and Structural Engineering, Shandong University, Jinan 250061, China; 3 Shandong, Energy Linyi Minimg, Group Co., Ltd., Linyi 276017, China

**Keywords:** three-dimensional coordination support, adaptability index, yielding–resisting sand column, structural design, mechanical properties

## Abstract

In view of the existing problems of stope roadways, which are difficult to maintain under the influence of high ground and mining-induced stresses, the structural characteristics and movement regularities of stopes surrounding rocks were analysed. Through the construction of a three-dimensional mechanical model of the coordination support of a stope, the adaptability index of the support in stope is presented, and its mechanism of operation is expounded. Yielding–resisting sand column (YRSC) sidewall-support technology with satisfactory compressibility and supporting strength was developed. The structure and actual mechanical properties of the YRSC were investigated through laboratory experiments, and the optimum ratio of filling materials was obtained. The good applicability of the load and deformation adaptability index of the three-dimensional coordination support in the stope and YRSC sidewall-support technology were demonstrated in practice at the No. 12306 working face of the Dongda coal mine. It was shown that the designed carrying capacity and compression of the sand columns satisfied the site requirements. The actual stress and deformation of the YRSC exhibited three stages: Slow growth at the initial stage, a large increase in the medium term, and a stable trend at the end. The adaptability index of the three-dimensional coordination support in the stope considers all bearing structure units of the stope as an interconnected whole, and the stability conditions of the stope roadway can be quantitatively described. The supporting effect of the YRSC is remarkable and can be applied to the construction of tunnels, bridge systems and other engineering fields.

## 1. Introduction

With the increase of mining depth and the intensity of extraction, the support and maintenance of roadways for the conventional pillar mining method are more difficult owing to the influence of various conditions, such as high ground stress and high-intensity mining. As a result, safety accidents, such as large deformation of roadways and roof falls, occur frequently, which results in difficulties in continuous tension about the processes of mining and excavating and disrupts the safe production in coal mines [1]. Under the background of a high-productivity and high-performance of a coal mine, in addition to the impact of the natural attributes of a typical “three high” environment of deep rock mass and the additional attribute of “strong disturbance” and “strong aging” for resource exploitation [2,3,4], the technology of roadways protection without coal pillars has attracted increasing attention owing to its advantages in terms of driving speed, coal recovery rate, and sustainable development [5,6]. The research of roadway stability and the design of scientific support technology are key to ensure safe and efficient mining.

The issues of a stope stability analysis and the applicability of different support technologies have been discussed by a number of scholars. Regarding a stope stability analysis, the influence parameters of stope stability in open stopes have been studied by Heidarzadeh and Diederichs [7,8]. Bagde [9] evaluated the effect of changes in the stope height on stope stability using empirical approaches and numerical modelling. Grenon and Hadjigeorgiou [10] used a three-dimensional joint-network model to study the stability of underground stopes by dividing rock blocks. The influence of coal pillar strength on stope stability has been studied by many researchers, such as Wattimena [11], Hou and Xie and so on. Hou and Xie investigated the mechanical characteristics of roadway-surrounding rock stress and its effects on the stability of roadway-surrounding rocks using the integrated research methods of numerical simulation, field measurement, and theoretical analysis, and put forward efficient methods of controlling the surrounding rock in deep roadways [12,13]. Jiang combined a multi-parameter monitoring method comprising electromagnetic radiation measurements, temperature, stress, and displacement monitoring to realise a different-spatial-scale and omni-directional monitoring of roadway-surrounding rock masses, which provided a new paradigm for understanding the stability characteristics of roadway-surrounding rocks [14]. Feng carried out a stability analysis of a stope in a gently inclined ore body with medium thickness, obtained many mechanical indexes that reflect the quality of engineering rock mass, and evaluated the stability of the stope [15].

Regarding the applicability of different support technologies, sidewall-support technology has evolved from conventional forms of timber packs, dense pillars, gangue belts, and concrete masonry walls to new methods, such as integrally poured sidewall filling and high-water quick-setting materials [16,17,18]. In recent years, based on the application of concrete-filled steel tubular structures in modern ground buildings and bridge engineering, the concrete-filled steel tubular support has been proposed as a new passive support form for controlling the stability of surrounding rocks in mine roadways. In this support, the inner concrete can prevent or delay the local bucking of the steel tube, and the steel tube can provide a confining effect on the concrete to enhance its strength and deformation ability. A number of experimental and analytical studies have been conducted on concrete-filled steel tubular columns [18,19,20,21,22,23,24,25,26]. Lee et al. focused on the evaluation of the axial load-carrying capacity of rectangular concrete-filled steel tubular columns with a high-strength steel slender section and the experimental and numerical results showed that the steel peak stress was affected by the lateral expansion of the crushed concrete [24]. Hu et al. devised an innovative test method to directly measure the load components of square concrete-filled steel tubular columns under axial compression and developed the existing stress-strain models [25]. Chang et al. presented a numerical study on the mechanical performance of concrete-filled steel tubular support and the numerical results showed that the interface behaviors of concrete-filled steel tubular support had an important influence on its mechanical performance and the friction coefficient of 0.4 was proposed [26]. Many experts have studied the mechanism and application of the concrete-filled steel tubular supports in deep and high-stress roadways, but its non-recyclable characteristics have limited its development to a certain extent [25,26]. 

The balance of mining-induced stress in the stopes is the result of the synergetic effect of the goaf, solid coal, and the support body. The instability of the surrounding rock in a stope and the failure process of the support are complex spatial mechanics problems, and the stress and deformation are two key influencing factors. Scholars have studied both the influencing factors and the evaluation methods of stope stability, as well as the technological and theoretical aspects of side supports in roadways, and have made great progress. In order to provide support criteria, scientifically and quantitatively, and to solve the influence of human factors in the evaluation process of traditional methods, it is still necessary to make a quantitative evaluation of the stability of the stope considering the two factors of stress and deformation. At the same time, the high labour intensity and low entry retaining rate of conventional support methods, as well as the complex support systems and high investment cost of new techniques, are beginning to hinder further utilisation and development of non-pillar mining technology, and it is imperative to find new ways of support that better accommodate different engineering fields [2,3,4]. 

In this manuscript, the load and deformation adaptability index of three-dimensional coordination support (TDCS) in the stopes was developed and the mechanism of its operation was expounded, a new type of yielding–resisting sand column (YRSC) sidewall-support technology with satisfactory compressibility and supporting intensity was developed. The experiments and field measurements indicate that, compared with the conventional sidewall-support techniques, the applicability indexes for TDCS give a good quantitative description of the stability conditions of a stope. The designed bearing capacity and satisfactory compressibility of the YRSC sidewall-support scheme meet the requirements of the field and this scheme has been proven to provide remarkable economic benefits and supporting performances, with strong potential for further expansion.

## 2. Theoretical Analysis of the Applicability Indexes for TDCS

Non-pillar mining technology is an important direction for the sustainable development of coal resources and an effective method to prevent major disasters and accidents in coal mines [27]. The carrying effect of a sidewall support is similar to that of a support under the action of a given load [28]. It must both accommodate the violent subsidence of the main roof in the early stage and carry the load of the main roof in the late stage. The practice has proven that the sidewall support does not need to prevent the subsidence of the main roof strata. Instead, a certain level of deformation in the roadway is more conducive to stope stability. The stability of the coordination support between the gangue, solid coal, and the supporting body in the goaf is one of the main factors that affect the success of roadside supports. The bearing system combines yielding properties with deformation control, so as to ensure the overall stability of the support system of the roadway-surrounding rock.

Based on the above analysis, this study puts forward effective measures for the quantitative evaluation of stope stability and makes a series of hypotheses: The roof is considered as homogeneous in the calculation process, and the roof is under the same stress. All of the immediate roof has collapsed in the goaf, and the sidewall support is bearing all the weight. According to the most dangerous state, the fracture along the coal wall is considered and the stress variation in the mining direction is not considered.

### 2.1. A Load Applicability Index for TDCS 

**Figure 1:**
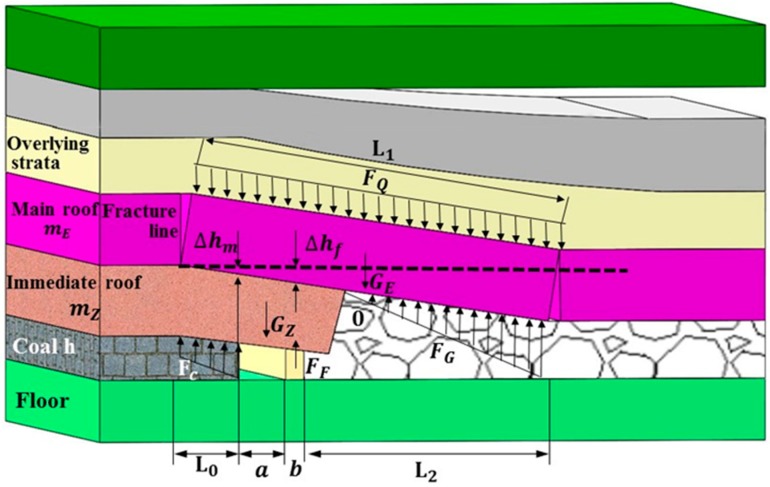
The three-dimensional coordination support (TDCS) mechanical model of the stope.

Based on the TDCS mechanical model of the stope, a load applicability index was established shown in Figure 1. In order to ensure system stability, the coal and roadside-support body, as well as the gangue in the goaf, must meet certain bearing capacity requirements. Through the mechanical analysis on the stope, it is needs to satisfy the following formula:

$$F_{C}+F_{G}+F_{F}\geq G_{Z}+G_{E}+F_{Q}$$

Then, the conditions for the system of roadways support technology, $F_{F},$ of the sidewall support are obtained:

$$F_{F}\geq G_{Z}+G_{E}+F_{Q}-F_{C}-F_{G}=A$$

where $F_{C}$ is the supporting force of the coal seam, $F_{G}$ is the supporting force of the gangue in the goaf, $F_{F}$ is the supporting force of the roadway side-support structure, $G_{Z}$ is the weight of the immediate roof, $G_{E}$ is the weight of the main roof, and $F_{Q}$ is the force of the overlying strata on the rock beams of the main roof.

At the same time, for a given load, the load of the immediate roof on the sidewall support can be approximately constant, and its value is approximated as $B=m_{z}\gamma_{z}f_{z}$, where B is the load on the sidewall support from the immediate roof, $f_{z}$ is the suspension top coefficient of the immediate roof, taken as 1.0, $\gamma_{z}$ is the average volume weight of the immediate roof, in kN/m^3^, and $m_{z}$ is the thickness of the immediate roof.

As discussed above, under the given deformation, the carrying capacity stability index of the sidewall support $F_{F}$ must also satisfy:

$$F_{F}\geq max\left\{A,B\right\}$$

### 2.2. A Deformation Applicability Index for TDCS

The weight of the overburden strata in the stope can be carried by the solid coal, supporting system, and the gangue in the goaf. However, the maximum permissible compression on all components of the system must stay within a reasonable level to maintain the stability of the stope structure.

At a working state with a given deformation, the final subsidence of the main roof is:

$$\Delta h_{A}=\frac{h-m_{Z}\left(K_{A}-1\right)}{L_{1}}\left(a+b\right)$$

To prevent sand column compaction, the maximum compression of the sand column must be satisfied:

$$\varepsilon_{max}=\Delta h_{A}-\sum \delta$$

where $\Delta h_{A}$ is the final subsidence of the main roof in metres, $K_{A}$ is the bulking coefficient of the immediate roof, generally taken as 1.30, $L_{1}$ is the lateral length of the main roof in metres [3,29], a is the width of the roadway in metres, b is the width of the sidewall support in metres, $\varepsilon_{max}$ is the maximum permissible compression of the sidewall support in metres, and ∑δ is the compression of the sidewall support through the roof/floor and the auxiliary support, as obtained from field measurement results.

To guarantee the stability of the roadway, the actual maximum subsidence of the sidewall-support system must stay within the maximum permissible deformation, which is defined by the respective carrying structures. First, to ensure that the sidewall-support system does not suffer instable failure, the maximum stress on the sidewall-support system, $\sigma_{f}$, must not exceed its ultimate strength:

$$\Delta h_{f}\leq \frac{h_{f}\cdot \sigma_{fmax}}{E_{f}}$$

where $\Delta h_{f}$ is the actual subsidence of the sidewall-support system in metres, $h_{f}$ is the height of the sidewall filler in metres, $\sigma_{fmax}$ is the maximum stress of the sidewall-support system in megapascal, and $E_{f}$ is the elastic modulus of the support.

Similarly, to accommodate ventilation, transport, and pedestrian traffic on the working face, the sectional compression of the roadway must not be larger than 25%. On this basis, the deformation capacities of the sidewall filler, subject to the limitations of the roadway size and of the coal side, are as follows. The ultimate deformation of the sidewall-support system:

$$\Delta h_{f}\leq \frac{h_{f}\cdot \left(L_{0}+a+\frac{b}{2}\right)}{3L_{0}+2a}$$

where $L_{0}$ is the distribution range of lateral abutment pressure in metres [3,29].

The permissible deformation capacity of the physical coal side:

$$\Delta h_{m}\leq \frac{h\cdot L_{0}}{4L_{0}+2a}$$

Then, the converted maximum permissible deformation of the support system is:

$$\Delta h_{f}\leq \frac{h\cdot \left(L_{0}+a+\frac{b}{2}\right)}{\left(4L_{0}+2a\right)}$$

As explained above, the permissible compression of the deformation applicability index for TDCS technology $D_{F}$ must satisfy:

$$D_{F}\leq min\left\{\varepsilon_{max},\;\Delta h_{f}\right\}$$

## 3. Structure and Mechanical Properties of YRSC

The key to the success in mining is to ensure the stability of the roadway during the process of subsidence of the overlying strata while meeting the deformation adaptability index of a stope three-dimensional coordinated support and reducing the cost of roadway protection as much as possible. In the initial stage of loading, the steel tube must quickly work as a temporary support with the assistance of single props. After that, it serves as a support, together with the filling aggregate in the steel tube. The sand particulate strengthens under the constraint of the steel tube, which in turn delays the buckling failure of the steel tube. This interaction significantly increases the overall carrying capacity and stability of the sand columns.

### 3.1. Structural Design of YRSC

**Figure 2:**
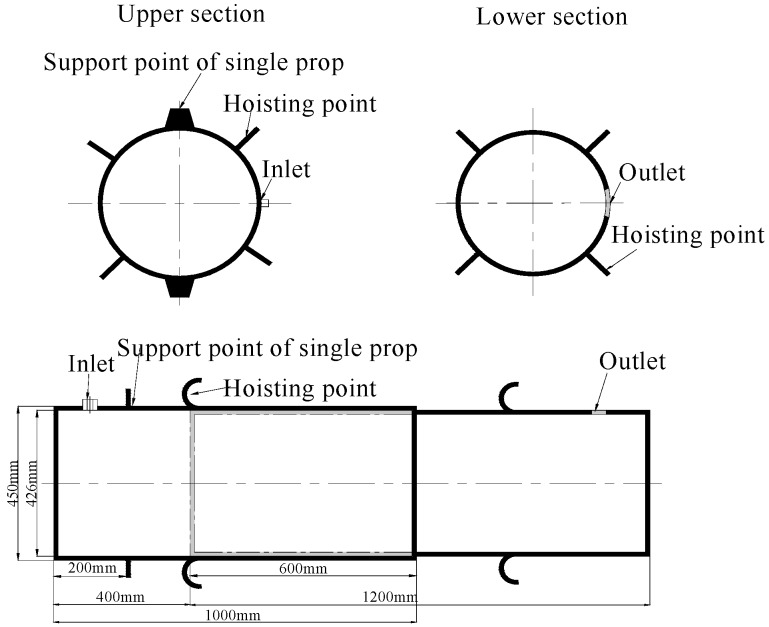
The structural design of yielding–resisting sand column (YRSC).

The sand columns were constructed from a 12-mm-thick Q345b seamless tube. The selection of the seamless tube should be based on the actual stope conditions. Each tube was divided into two sections to accommodate the height variation of the roadway. The lower section measured Φ426 × 10 mm and extended 1.2 m in length and the upper section measured Φ450 × 9 mm and extended 1.0 m in length. The two sections were nested and filled with filling materials. The actual structure of the sand column is shown in Figure 2. A filling inlet was welded to the upper section, while an outlet was welded to the lower section. The two sections overlapped for no less than 500 mm. To facilitate mould erection, the lifting points were welded to the sand columns so that the upper section of the mould could be erected to the roof with single props. For this purpose, two symmetrical single-prop support bases were attached on the upper section of the mould, 200 mm from the top, to support the single props and accommodate the initial support load of the sand columns during its installation. After the sand columns were installed, the sand columns were supported with an anti-inversion rope. The anti-inversion rope was fixed diagonally to ensure that the roof and sand columns were in tight contact. A grout injector was used to fill the sand columns. The clamps were fabricated to connect the grout pipe to the sand inlet. A vibrator was installed outside the sand columns to compact the grout.

### 3.2. Mechanical Properties of YRSC

**Figure 3:**
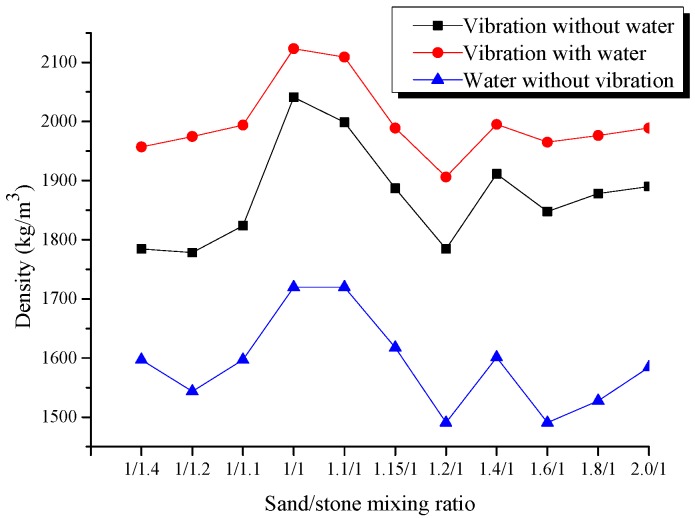
The influence of the different filling materials ratio.

A 4000t laboratory multifunctional electrohydraulic servo harmony loading system was used to load the sand columns and thus examine their ultimate carrying capacity under axial stresses in different conditions. The filling material was a mixture of medium-grained fluvial sand and stone chips with a 5–10 mm particle size. The mixing ratio influences the density of the filling materials directly and hence affects the stability of the sand column. An experiment was performed to examine how water and vibrations affect the density of the filled sand columns. The vibration is carried out with a special vibration device customized for coal mines, and the water flow is applied until the upper surface of the filling aggregate is not obviously settled. The water flows out through the lower sand outlet, which only removes air and ensures compactness. Under the action of pressure, the water gradually flows out, ignoring the influence of the residual water on the strength of sand column. The result is given in Figure 3.

In the experiment, the same stones were used as in the field, and the common proportion of the secondary aggregate was used. The proportion of small stones (5–20 mm) and medium stones (20–40 mm) was 5.5:4.5. When the sand/stone mixing ratio increased from 1:1.4 to 1:1, the overall density of the filling material increased slightly; but when no water was added, the discreteness of the density fluctuations was large. The amplitude variation was smaller than without water. Under the vibration, the density of the filling material displayed roughly the same variation with and without the water under all the filling material ratios. When the water was added, the density of the sand column filler was obviously larger than that obtained when no water was added and reached its maximum when the mixing ratio was 1:1. Hence, 1:1 was selected as the optimum ratio of the filling materials.

**Figure 4:**
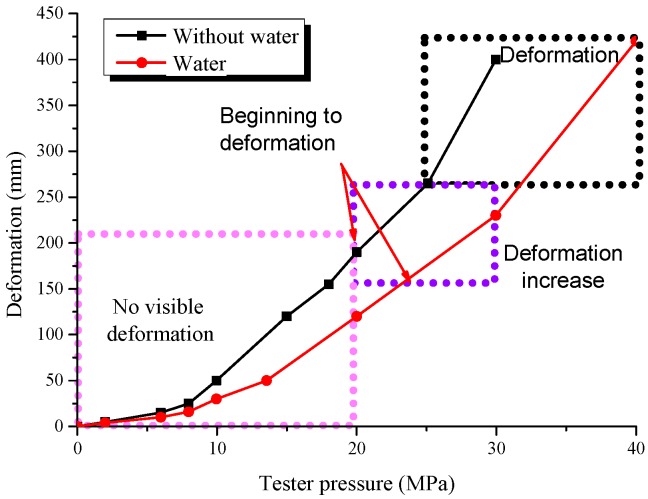
The carrying capacity experiment.

The carrying capacity experiment of the sand column was made using the selected optimum ratio of filling materials. The stress sensor was installed in the middle of the sand column through the sand outlet. The results are given in Figure 4. The results showed that the experiment can be divided into three phases. First, there was no obvious deformation of the sand columns as the testing machine pressure increased. When the pressure was further increased, the sand columns entered the second phase, where they began to be damaged at the bottom. When no water was added, the bottom of the sand column began to deform at a stress of 20 MPa. This deformation appeared to further intensify with increasing pressure. When water was added, the sand column began to deform when the stress increased by approximately 30% from 20 MPa, and their carrying capacity increased notably. At the same pressure level, their compression was smaller than when no water was added. For instance, at 20 MPa, their compression was 190 mm when no water was added, but was reduced to 120 mm when water was added. The reduction was almost 70 mm. In the third phase, at 30 MPa, the deformation and failure in the lower part of the sand columns continued to increase. The compression of the sand column increased sharply. The sand outlet of the sand column, which is the weakest point of the steel tube, began to rupture until failure. The final failure pressure of the sand columns was 40 MPa when water was added and was approximately 33% higher than when no water was added.

Overall, when the water was added and the vibration was applied, the compression of the sand columns was significantly lower and their carrying capacity was much larger. As analysed above, this study decided to use the 1:1 sand/stone mixing ratio and to construct the sand columns by using filling containing water and by applying the vibration.

## 4. Field Test and Field Monitoring

In order to verify the field application effect of the load and the deformation adaptability index of the three-dimensional coordination support and the new type of yielding–resisting sand column, and facilitate the further improvement and promotion of this technology, the on-site monitoring of Dongda 12306 working face in Tengzhou was carried out.

### 4.1. Mining and Geological Conditions

The No. 12306 working face of the Tengzhou Dongda coal mine has a strike length of 1013 m and a dip length of 219 m. The average seam thickness is 1.8 m. The structure is quite stable. The immediate roof consists of mudstone with an average thickness of 0.8 m. The main roof consists of fine-grained sandstone with an average thickness of 14.85 m and the lithology is tight. The immediate floor consists of limestone with an average thickness of 3.34 m and the bedding is quite thick. The geological condition of the working face is relatively simple and no fault has developed.

**Figure 5:**
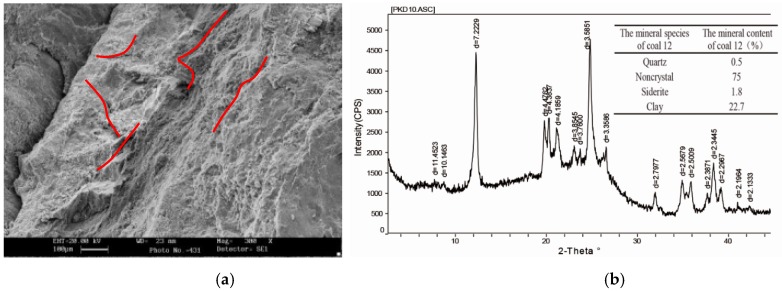
Scanning electron microscopy and X-ray diffraction analysis. (**a**) SEM image magnified 500 times. (**b**) X-ray diffraction pattern of coal sample.

To understand of the mineral composition and microstructure of the coal seams, coal samples collected from the site were analysed by scanning electron microscopy (SEM, JEOL.Ltd., Akishima, Japan) and X-ray diffraction (XRD, Rigaku, Akishima, Japan) [30,31]. The results are shown in Figure 5. Further examination revealed that the coal body features developed microfissures, good connectivity, broad fissures, and a poor microstructure. These features make it easy for fissures to expand and connect to each other under high mining stresses, which can significantly compromise the stability of the surrounding rock. Also, the non-crystal content of this coal mine is as high as 75%, while the quartz content is merely 0.5%. The high clay proportion can influence the coal–rock and roadway stability directly. Therefore, the conventional pillar retaining method not only results in resource waste and mining discontinuity, but also involves considerable safety risks. Hence, to attain the high-productivity and high-performance target, this study planned to adopt a new sand column scheme on the No. 12036 working face.

### 4.2. Analysis of the Load and Deformation Applicability Indexes

According to the existing geological data of the No. 12036 working face of the Dongda coal mine, a systematic analysis was conducted on the applicability of TDCS technology with sand columns using the findings described above. Combined with the existing geological data and field measurement of this mine, the distance of the fracture line into the coal wall, $L_{0}$, is 3.79 m; the lateral length of the main roof, $L_{1},$ is 16 m; if the gob is fully filled with gangue, the gangue support distance $\mathrm{is}\;L_{2}$ 8.76 m; the design section size of the roadway is 3 m × 2.2 m (width×height); the average unit weight of the immediate roof is 22,840 N/m^3^; the average unit weight of the main-roof micropsammite is 25,600 N/m^3^; the gangue carrying capacity, $F_{G}$, is 7 MPa; the elastic modulus of the sand column, $E_{f}$, is 0.14 Gpa [32,33,34]; the thickness of the overlying rock strata is 600 m; the average unit weight of the overlying rock strata is 26,400 N/m^3^, the height of the caving zone is 14.35 m. By substituting the geological and technical parameters of this working face in the following equation:

$$F_{F}\geq G_{Z}+G_{E}+F_{Q}-F_{C}-F_{G}=A$$

$$F_{F}\geq m_{z}\gamma_{z}f_{z}=B$$

The maximum carrying capacity of the sand columns reached 40 MPa during the experiment, corresponding to the force of 6,361,537 N. As discussed above, the carrying capacity stability index of the sidewall support meets the requirement, which satisfies the load applicability index for the TDCS in the stope.

With regard to the deformation applicability index of the TDCS in the stope:

$$\Delta h_{A}=\frac{h-m_{Z}\left(K_{A}-1\right)}{L_{1}}\left(a+b\right)=0.34;$$

$$\Delta h_{f}\leq \frac{h_{f}\cdot \sigma_{fmax}}{E_{f}}=0.514m;$$

$$\Delta h_{f}\leq \frac{h_{f}\cdot \left(L_{0}+a+\frac{b}{2}\right)}{3L_{0}+2a}=0.727m;$$

$$\Delta h_{f}\leq \frac{h\cdot \left(L_{0}+a+\frac{b}{2}\right)}{\left(4L_{0}+2a\right)}=0.597m;$$

The final failure pressure of the sand columns was 40 Mpa and the final failure deformation of the sand columns was 400 mm when the water was added. By comparison, on the premise of meeting the support strength, the deformation of the sand column meets the deformation adaptability index, that is to say:

$$D_{F}\leq min\left\{\varepsilon_{max},\;\Delta h_{f}\right\}$$

It can be seen that the mining condition satisfies the deformation applicability index for the TDCS in the stope, and the sand column is strong enough to accommodate the gob-side entry retaining (GSER) on the site.

### 4.3. Support Monitoring and Effect Analysis

**Figure 6:**
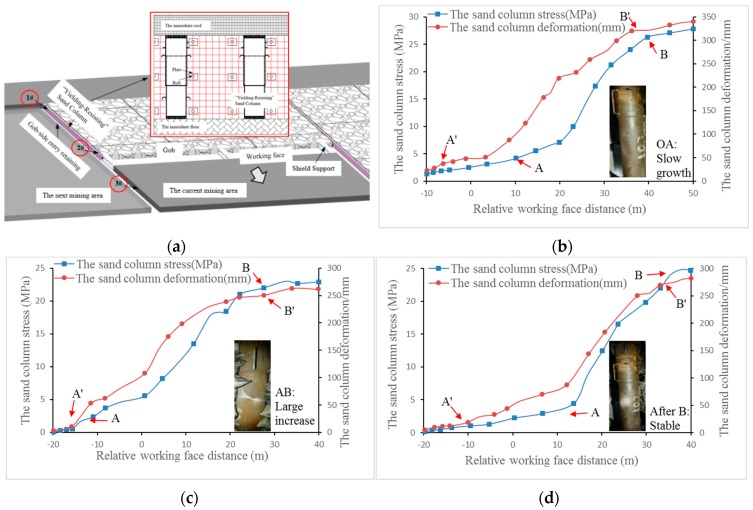
The stress and deformation field monitoring of the sand columns. (**a**) Layout of stope, (**b**) Measuring point 1#, (**c**) Measuring point 2#, (**d**) Measuring point 3#.

To investigate how the sand columns function on the site, three representative measuring points were selected to record the load and deformation variations in the sand columns during the construction process. According to the actual situation of the No. 12306 working face, the sand columns were constructed from 12-mm-thick Q345b seamless tubes. The measurement results are given in Figure 6. As the working face advanced, the deformation of the sand columns at each measuring point began to increase gradually from the rear of the working face to the range of −5 m to −15 m (as shown in the OA′ section in Figure 6).The sand columns tended to be stable when they were 40 m away from the working face (as shown in Figure 6 after point B′). The growth rates of the stress and deformation at each measuring point experienced three stages: Slow growth at the initial stage; a large increase when approaching the working face; a stable trend away from the working face. The sand column at measuring point 1#, which was located close to the open-off cut of the working face, was significantly subjected to mining pressure. At the beginning of the measurement, it received substantial pressure, which resulted in considerable compression and initial stress. Finally, when it was 40 m away from the working face, its stress and deformation stabilised at approximately 27 MPa and 341 mm, respectively. The sand column at measuring point 2# was located in the middle of the working face. At the beginning, the roof pressure increased slowly. The initial compression of the steel tube was smaller than at measuring point 1#. When it finally stabilised, its stress and deformation were approximately 23 MPa and 260 mm, respectively. The sand column at measuring point 3# was close to the stop line. Affected by the mining stress, the final loads and the compression were larger than at measuring point 2# but smaller than at measuring point 1#, and they finally stabilised near 25 MPa and 285 mm, respectively.

According to the measurement results, the sand columns have good compressibility and the maximum compression was 342 mm. Meanwhile, the maximum stress exerted on the sand columns was no more than 30 MPa, which is much smaller than the 40 MPa measured in the mechanical experiment on the carrying capacity of the sand columns. As demonstrated above, the designed carrying capacity and deformation of the sand columns are suitable to maintain good supporting resistance within a controllable extent of the deformation, and GSER can be applied to the Dongda coal mine.

**Figure 7:**
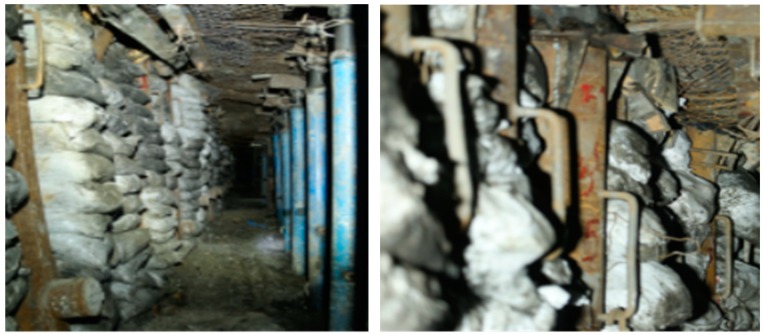
Field application results.

The adaptability index of TDCS technology in a stope and GSER with sand columns have achieved satisfactory results on the No. 12306 working face, as shown in Figure 7. The working face successfully retained the roadway for 700 m, from where 12491t of coal is recovered, and has produced an economic benefit of 8 million yuan. The sand columns provided satisfactory carrying capacity. The application of this technology reduces the time needed for preparing working faces and releases the tension relationship between gateway excavating and the coal mining operation. Furthermore, the sand columns can be reused and their recycling rate on working faces can reach 85%. This gives benefits to the use of high-performance, low-cost sand columns as sidewall fillers for GSER and provides rich theoretical results for the promotion and popularisation of GSER technology with sand columns.

## 5. Conclusions

The load and deformation adaptability index of the three-dimensional coordination support in stopes was developed and the mechanism of its operation was expounded in the present work. A new type of yielding–resisting sand column sidewall-support technology with satisfactory compressibility and supporting intensity was developed. The following conclusions can be drawn based on the reported study:

(1) Based on the in-depth analysis of the structural characteristics and movement regularity of stope-surrounding rocks, the TDCS mechanical model of the stope was established. Additionally, the adaptability index of TDCS in the stope was presented, and a new type of YRSC sidewall-support technology with satisfactory compressibility and supporting intensity, which guarantees high yield and efficiency in coal mines, was developed.
(2) In view of the two major factors of stress and deformation affecting the stability of the surrounding rock in a stope, and by combining the interconnected whole with the gangue, solid coal, and the supporting body in the mining area, the theoretical analysis and field measurements show that the effect of the application of the adaptability index of the stope is good.
(3) The support structure and mechanical properties of the YRSC were studied, and the optimum ratio of filling materials and the maximum supporting intensity were obtained. The field measurement revealed three loading phases for the stress of sand columns, from a slow increase to rapid elevation and eventually to stability. The maximum stress and compression were approximately 30 MPa and 342 mm, respectively, both lower than the experimental values. The designed carrying capacity and compression of the sand columns are suitable for maintaining satisfactory supporting resistance within a controllable level of deformation and meets the requirements of the TDCS in the sidewall of the stope.

Although the existing research has made some progress, due to the complex geological conditions of coal mines, it is still necessary to further carry out the coordinated control research of surrounding rock stress and deformation in combination with complex and diverse engineering geological conditions. At the same time, the application of yielding–resisting sand column sidewall-support technology is still in the preliminary stage, especially for its theory and simulation research which need to be further studied.
